# Breaking the Stigma in Mental Health Nursing through High-Fidelity Simulation Training

**DOI:** 10.3390/nursrep13040132

**Published:** 2023-11-03

**Authors:** Agustín Javier Simonelli-Muñoz, Diana Jiménez-Rodríguez, Oscar Arrogante, Fernando Jesús Plaza del Pino, Juana Inés Gallego-Gómez

**Affiliations:** 1 Department of Nursing, Physiotherapy and Medicine, University of Almeria, 04120 Almería, Spain; sma147@ual.es (A.J.S.-M.); djr239@ual.es (D.J.-R.); juanai@ual.es (J.I.G.-G.); 2 Department of Nursing, Faculty of Nursing, Physiotherapy and Podology, University Complutense of Madrid, 28040 Madrid, Spain; oarrogan@ucm.es

**Keywords:** high-fidelity simulation training, mental health nursing, nursing education, qualitative research, social stigma

## Abstract

The social stigma toward individuals with mental health problems is habitual among nursing students, which can lead to poor quality of health care services for patients with mental illnesses. The purpose of the present study was to learn about nursing students’ perceptions of providing care to patients with severe mental disorders before and after participating in a simulated student clinical case. A descriptive qualitative study was conducted through 39 interviews. The difficulties expected by the students and their perceptions about patients were explored before the simulation training. Their perceptions about the use of clinical simulation for learning about the adequate management of these patients were analyzed afterwards. Results: Before the simulation training, the students assessed the case as being complicated, expressed their lack of specific training, and felt fear and insecurity, thus reproducing the stigma towards mental health patients. After this training, they positively valued the usefulness of the clinical simulation for gaining confidence and overcoming the stigma. Discussion: The use of high-fidelity simulation offers nursing students the opportunity to approach patients with mental health conditions, overcoming their fears and normalizing mental disorders. Simulation training allows nursing students to analyze the reasoning of clinical judgment and to detect the influence of previous prejudices about mental illness in their clinical decision. This study was not registered.

## 1. Introduction

Mental disorders are presently one of the major problems in public health [1]. In the last decade, an increase of 13% has been observed in mental disorders and substance abuse. These disorders result in living with a disability for 1 to 5 years, with depression being one of the main causes of this disability. Also, the fourth cause of death in individuals aged between 15 and 29 years old is suicide [2]. Despite the advancements in some countries, patients with mental health problems suffer severe violations of human rights, discrimination, and stigma [2].

According to theorists such as Goffman [3], the concept of stigma has a double connotation. On the one hand, it is an attribute that turns the other into a different individual which relates them to a series of undesirable characteristics or negative stereotypes of the person. On the other hand, it is considered a socially constructed product of rejection or disapproval by the social environment with its corresponding negative responses or unwanted effects in terms of behavior that person [3].

Mental health-related stigma is often grounded in stereotypes that persons with mental health issues are dangerous (unpredictable, violent), responsible for their mental health issue, cannot be controlled nor recover, and should be ashamed [4]. The phenomenon of stigma towards mental health patients is not restricted to specific communities or countries [5], and it is common among primary care professionals [6]. Consequently, this phenomenon can therefore negatively affect the care provided to the mentally ill user [7].

### 1.1. Background

Many nursing students hold negative assumptions about mental health practice, with fearful stigma and stereotypes [8]. They can often lack confidence in communicating with people with mental disorders and may be afraid to do or say something harmful [9]. However, discriminatory and negative attitudes can be addressed in specific anti-stigma interventions and ideally incorporated into mental health nursing education among undergraduate nursing students from the beginning of professional training [10]. In this sense, a systematic review concludes that mental health-specific training seems to improve nursing students’ perceptions toward mental health, highlighting that clinical placement underpins theory, leading to a decrease in negative attitudes and stigma regarding mental health [11]. Specifically, a recent review and meta-analysis support the effectiveness of simulation training in mental health nursing education [12].

Modern stereotypes still present the mentally ill as at fault, unpredictable, and violent [13], and observational studies have indicated that health professionals, including nurses, are often part of the stigma [6,13]. However, many people who suffer from mental health problems will receive care and attention from nurses at different stages of care throughout their lives. For this reason, there is a strong need to re-enforce education programs [14]. These programs will allow nursing students to tend to the needs of these patients and to offer a comprehensive approach to the care of mentally ill patients [15].

Nursing departments at many Spanish universities now include a specific course about psychiatry and mental health. However, some nurses believe that greater exposure to problems associated with mental health is needed, not only in communities but also in health sciences faculties [5,16]. However, a general psychiatry course during the nursing degree may not, by itself, change the biased point of view about mental health [7]. Therefore, they propose re-enforcement through exposure and interaction with patients with mental disorders [7,17].

In this sense, nursing students enrolled in the mental health course and practical sessions have a diversity of pre-conceived ideas. Professors play a central role in the identification and development of psychoeducational strategies to address the concerns of the students and to increase their interest in the mental health speciality [18]. Clinical simulation is an excellent medium for learning and evaluating skills, both technical as well as non-technical, and in the field of nursing, the use of this approach has been increasing to help students acquire basic competences [19]. Specifically, our university added training activities to its study plan, in which clinical simulation scenarios play a relevant role in the teaching and evaluation of competencies, thus ensuring the acquisition and practical application of the professional qualities required in nursing [20].

Consequently, it is necessary to train nursing students on mental health through clinical simulation sessions. The objective for their use is for the students to acquire specific skills and competences which will allow them to improve their view of mental health patients, minimizing their suspicions and fears and thus allowing them to improve the nursing attention and care of individuals with mental disorders.

### 1.2. Rationale and Aim

Although specific anti-stigma interventions are recommended in undergraduate nursing students [10] and simulation training is effective in mental health nursing education [12], most previous studies have mainly adopted a quantitative approach, and the few qualitative studies available have mainly the nursing students’ perceptions only after simulation training, without their pre-conceived ideas about mental illness. Moreover, few studies have included standardized patients who played the role of severely mentally ill patients.

Therefore, the main aim of our study is to learn about nursing students’ perceptions of providing care to patients with severe mental disorders before and after participating in high-fidelity simulation training using standardized patients.

## 2. Materials and Methods

### 2.1. Study Design

We conducted a descriptive qualitative study in order to explore the experiences and perceptions of our participants [21], considering their circumstances and their own points of view [22]. Moreover, in our case, we aimed to explore the perceptions of nursing students before and after participating in a simulated case of a patient with a mental disorder. The recommendations of the COREQ guide for qualitative research reporting [23] were followed.

### 2.2. Sample and Environment

This study involved students in the last year of their nursing degree at a public university in southern Spain. According to the European Qualification Framework, the 4-year nursing degree in Spain meets level 6. These students had taken the third-year course, nursing in mental health, and took part in clinical simulation scenarios related to mental health within the context of the Practicum IV-V course in their last (4th) year. Voluntary participation was offered to all of the students (56 students). The study was conducted between October and November 2021.

### 2.3. High-Fidelity Simulation Procedure

A high-fidelity clinical simulation scenario was developed about a patient diagnosed with borderline personality disorder, according to DSM-5-TR [24], and admitted to a long-term hospital residency unit; the patient’s name has been changed (Table 1). This clinical case represented in the simulated scenario was designed by three registered nurses specialized in mental health with more than ten years of experience in patient care for the mentally ill. The participation of students during the simulation was evaluated according to the nursing activities associated with the appropriate Nursing Interventions Classification (NIC) [25]. See Table 1 for the resolution of this simulated clinical case.

Both the NIC interventions and the nursing activities served as a guide to discuss the performance of the students during the debriefing phase. Even though the students were familiarized with the NIC interventions and the nursing activities related to subjects covered in their previous courses about the theme, they were not previously informed that their performance would be evaluated based on them.

The main learning objective of the simulation scenario was for students to learn how to provide good nursing care to a patient with a mental disorder by utilizing nursing competences associated with emotional support and relaxation through the teaching methodology of clinical simulation. To achieve this objective, a total of 21 clinical simulation sessions were conducted. The students were divided into work teams composed of 2–3 nursing students for each simulated clinical case. Thus, all the students were able to participate in the simulated scenario designed. All the clinical simulation sessions followed the Best Practices Standards proposed by the International Nursing Association of Clinical and Simulation Learning (INACSL) during its four phases [26]: pre-briefing, briefing, development of the simulated scenario, and debriefing. Two university professors, with more than ten years of experience in clinical simulation methodology, carried out and supervised the four phases of this methodology recommended by the INACSL. It must be pointed out that in the pre-briefing phase, a psychologically safe context was promoted, following the recommendations by Rudolph et al. [27]. Also, in the debriefing phase [28], the tool used was the GAS (gather, analyze, and summarize) technique proposed by Phrampus and O′Donnell [29].

Below is a description of the phases developed during the high-fidelity clinical simulation sessions:

- Pre-briefing: Two weeks before the simulated scenario sessions, the students were provided with information to establish a psychologically safe environment and to consolidate the learning process [27]. Thus, a document with a brief description of the simulated scenario was provided to each student so that they could search and plan its resolution through an evidence-based approach. This information included details such as medical history and state of health (young man who was recently admitted due to a mental disorder and who was angry for his admission).
- Briefing: the clinical scenario was put into context through a brief presentation of the clinical case background.
- Simulation scenario: The clinical scenario was as real as possible. This scenario aimed to provide emotional support and to use relaxation techniques with a young patient who had just been admitted due to a mental disorder. For this, 5 main nursing activities were related to the corresponding Nurse Interventions Classifications (NIC). These NICs were only used as a guide, as the evaluation of the students’ performance was broader. During the clinical simulation session, video cameras and microphones installed in the simulation room were utilized.
- Debriefing: After the end of the simulation scenario, it was analyzed and discussed [28], with this phase structured with the debriefing tool GAS (gather, analyze, and summarize). Following this, the nursing students discussed and analyzed the practices based on the best available evidence related to the clinical case, promoting anin-depth reflection and analysis of the nursing activities that were well conducted, the mistakes, and those that needed to be improved during the clinical simulation session. The nursing professors trained in the clinical simulation methodology provided feedback to the students about their clinical performance.

### 2.4. Data Collection

Semi-structured interviews with open-ended questions were given to each participant in two phases: the first phase before taking part in the high-fidelity clinical simulation (pre-CS) and the second phase after the last simulation session (post-CS). The interview guide was designed and validated by the study researchers in agreement with the theoretical categories related to the theoretical foundations of the debriefing phase in the clinical simulation methodology [29].

The pre-CS phase was centered on delving into the expected difficulties with the case according to the students and the perceptions of the students concerning the patients with mental health disorders. The post-CS interview phase was oriented toward the use of high-fidelity clinical simulation for learning how to manage patients with mental disorders. Below, Table 2 shows the interview guide.

All of the interviews were conducted in a room that was set up for this at the university, seven days before the start of the first simulation session (pre-CS phase) and seven days after the last session (post-CS phase). The interviews lasted an average of 25 min per phase. The interviewer was a member of the research team who did not participate in the simulation sessions, thus avoiding possible biases. All of the interviews were recorded with the verbal consent of the participants.

### 2.5. Data Analysis

The interview recordings were transcribed and reviewed by two researchers to ensure that the transcription was correct. The data were stored, managed, classified, and organized with help from the qualitative data analysis software ATLAS-ti 8.0 (Scientific Software Development GmbH, Berlin, Germany). We followed the steps for adequate descriptive qualitative analysis [30]: (1) transcribing and sorting the data, (2) giving codes to the initial data obtained from interviews, (3) adding comments/reflections (memos), etc., (4) trying to identify similar phrases, patterns, themes, relationships, and sequences, (5) taking these patterns and themes to help focus the next wave of data collection, and (6) gradually elaborating a small set of generalizations that cover the consistencies you discern in the data. Thus, an explicit description of the perceptions of the students about the experience was obtained. The categories identified were initially aligned with the subjects of study proposed in the two phases of the interview. Two researchers, who are experts in qualitative research, developed the entire process of obtaining categories and subcategories independently, ending the process with the sharing of both and a consensus on the final decisions of the analysis.

### 2.6. Ethical Considerations

The research protocol was approved by the Research Ethics Committee from the Department of Nursing, Physiotherapy, and Medicine at the University of Almería (EFM 75/2020). To guarantee anonymity and confidentiality, a code was assigned to each participant. In all of the cases, an informed consent form was read aloud and explained to the students, which detailed the aim of the study and the acceptance of the student to participate in the study, with the students who wanted to participate signing the form.

### 2.7. Qualitative Rigor

The quality of our study was evaluated according to the criteria of Lincoln and Guba [31]. They assessed the credibility, transferability, and confirmability of the research. Data were triangulated between the researchers involved in the analysis, and independent researchers reviewed the analysis process to ensure credibility. Transferability was guaranteed by describing the research setting, as well as the participants, context, and method. Confirmability was achieved through the presence of variability in participants’ experiences; reading and analysis were conducted by each researcher independently, contrasting and then agreeing on emerging themes and subthemes.

## 3. Results

A total of fifty-six students in the last year of their nursing degree attended the high-fidelity clinical simulation sessions, of which thirty-nine accepted to participate in the study (response rate of 67.5%). Those who did not participate were due to disinterest, lack of time, or other reasons. Most of the participants were women (76%), with a mean age of 23 years old (mean = 23.30; SD = 6.932).

As a preliminary exercise, at the start of the pre-CS interview, the participants were asked to order various simulation scenarios centered on patients from different medical specialities, including the scenario to be analyzed, according to their participation preference; more than half of the informants placed the mental health patient case in the last place. The reasoning behind this decision appears in the presentation below of the different categories and subcategories. Regardless of where they placed the mental health patient case in terms of their preference, all thirty-nine participants completed the two interviews.

Table 3 presents a summary of the subcategories identified, according to the different questions asked to the students.

The six main categories that appeared from the open-ended question and their corresponding categories, supported by the narratives of the participants, are described below, differentiating the pre-CS and post-CS categories. It should be noted that all the interviews were performed in Spanish and the quotes were translated into English by a bilingual native speaker, thus avoiding the loss of important semantic details.

### 3.1. Pre-CS Categories

During the pre-CS interview phase (before entering the simulated scenario), the students pointed out the difficulties that they thought they may encounter; they believed the case to be complicated and did not think they had sufficient training. We can find the social stigma suffered by these patients with mental health conditions in the perceptions of the majority of the students, although, for other students, they were considered as just another patient. In general, before taking part in the simulation, they started feeling fear and insecurity.

#### 3.1.1. What Difficulties I Will Find

Together with the previous exercise in which they arranged the possible scenarios according to their participation preference, among which we find a pediatric scenario, another with a patient under hemodialysis, and one involving surgery, they were asked to justify their answer and to express the difficulties they believed they would find in this specific scenario. Most of the students placed the case addressed in the present study in last place, as they perceived that this scenario included diverse difficulties, which will be described below.

- Complex case:

Many informants mentioned the complexity they perceived in the case due to the protagonist being a mental health patient. It was noted that their comments were strongly determined by their own beliefs and/or prejudice against these patients.

*Treating a patient with a mental illness is difficult in many aspects, some of them are more susceptible to the usual and could think that it hurts them instead of helping them*.

(S9)

*It is difficult to make patients with personality disorders collaborate, it is quite a challenge*.

(S18)

*Communication with this type of patient can be complicated*.

(S31)

*In my opinion, it is complicated, as you are never 100% sure about how the patient will react*.

(S3)

- Not knowing:

Students frequently expressed their lack of competences/skills for addressing a mental health patient, despite having taken a specific course during their nursing studies. They fundamentally referred to non-technical or socio-emotional skills such as communication skills and emotional management.

*Not knowing how to address the situation when Juan loses it*

(S1)

*Knowing how to interact with him and be part of the negotiation*.

(S16)

*Knowing how to address a patient with a mental illness is foreign to me, and I think that a lot of professional experience is needed for addressing this type of patient*.

(S18)

*Not knowing how to address or calm the patient due to a lack of knowledge or expression techniques for talking with patients with mental illnesses*.

(S30)

#### 3.1.2. Perception of the Patients with a Mental Disorder

Among the nursing students’ perceptions about mentally ill patients, we found that some students had pre-conceived ideas (such as prejudices and social stigma), while others perceived these patients as any other, without pre-conceived or prejudiced assessments.

- The prejudices; the stigma:

Within the comments from our informants, we observed some pre-conceived ideas, and in some cases, the social stigma suffered by people with mental health problems is very present. This is an important finding, as the beliefs or perceptions determine the possible future behavior toward these patients.

*Emotionally-unstable individuals, if I could define them with a single word, it would be ‘unpredictable’*.

(S22)

*Non-collaborative, non-communicative*.

(S11)

*Emotional liability, extreme behavioural changes…*

(S8)

*Abnormal beliefs, difficulty in thinking clearly, sadness, anxiety, mood changes*…

(S17)

On the other hand, despite stating that they were not wellversed in these individuals, many of the participants talked about a theory or analysis of these patients (pre-conceived ideas).

*They are unique, they are scared about their true nature or they fear things that other people, depending on their past, would not fear, they have difficulties we are not able to see and/or be empathetic with*…

(S5)

*They have very defined routines in their head and it is very difficult to change them*.

(S12)

*People who have suffered and need a lot of support. They are warm and grateful*.

(S32)

*They do not recognize their illness, they live in a reality that does not exist and believe that everyone else is trying to harm them and do not care about them*.

(S18)

Also, many students believed that these patients are found outside of the established norms, cataloguing them as strange individuals.

*Those who are outside of what is considered in society to be normal*.

(S8)

*They are people who live in the fringes of society, they are different from the rest*.

(S9)

*The typically strange and extravagant person who lives in his or her own world*.

(S29)

- Like another patient:

However, we also found a minority of students who perceived these individuals as any other patient, without pre-conceived notions or prejudiced assessments; they are Please confirm if the bold is unnecessary and can be removed. The following highlights are the same. Simply another patient to provide care for.

*It depends on the disorder, you cannot define a person with a characteristic if you do not know his or her history*.

(S2)

*As any other normal person who has an illness, and needs someone to listen to them, to feel comfortable*.

(S19)

*For me, there is nothing that defines them, they are just like the other people with an illness, another patient*.

(S25)

*People with a pathology, just as others have hyperthyroidism. I believe that we should not have prejudices because they are like any other person with or without a pathology*.

(S30)

#### 3.1.3. Emotions Felt with Regard to a Patient with a Mental Disorder

Among our informants, we found a predominance of negative emotions regarding contact with a person with a mental disorder, such as fear or insecurity, which could make approaching the patient more difficult, as well as determining the therapeutic relationship that could be established.

- Fear:

The students expressed that they felt fear when they were told they would have to interact with a patient with a mental disorder, and this emotion was associated with the lack of knowledge about these individuals, as well as their lack of experience.

*As we are dealing with a mental health patient, we always feel more fear and insecurities*.

(S5)

*I have never provided care for them but maybe fear that they will not like what you say and challenge me*.

(S18)

*Fear of not knowing how to communicate so that he understands everything you explain and does not misinterpret anything*.

(S22)

- Insecurity:

Strongly associated with the previous feeling, the students felt insecure about the relationship with these patients, in many cases due to the perception of lack of skills and specific training in mental health.

*The uncertainty of dealing with a person with non-controlled mental problems*.

(S25)

*Uncertainty about how he will react when you have to perform a technique with him, I see them as unpredictable individuals, and I feel unsure, in case I don’t know how to deal with them*.

(S10)

*Distress for wanting to help but lacking the necessary tools for it*.

(S28)

*I don’t think they are aggressive, but they seem to be unpredictable, and I’m unsure in case I don’t know how to deal with them*.

(S36)

#### 3.1.4. What Is Lacking in My Training

Among the deficits in their training, the students highlighted both the communication skills as well as the lack of clinical experience and in other cases, did not clearly identify their training needs.

- Communication skills:

The students highlighted the need to be able to maintain adequate communication with their patients and considered the lack of this ability.

*I need to know how to communicate, I don’t think I’ll be able to connect*.

(S28)

*Communication is the main problem that he doesn’t understand what I say, and despite understanding it, he does not agree*.

(S39)

- Clinical experiences:

Despite being enrolled in the final stage of their nursing degree education, the students recognized their lack of clinical experience in mental health as one of their main handicaps.

*The main difficulty is borderline personality disorder, as you have to know how to deal with it at all times, and I’ve never done that*.

(S18)

*I lack the necessary experience to know how to address it correctly*.

(S32)

- Unspecified training:

In some cases, our informants were aware of their lack of training in this field, although they were not able to define their deficiencies.

*He is patient with mental health condition, with them, you have to have certain skills to be able to manage them*.

(S4)

*With patients with a physical illness, it’s easier, you learn a technique, and that’s it, but with mental illnesses, you need other, more complicated things, but I don’t know how to explain them*.

(S27)

### 3.2. Post-CS Categories

Once the simulated scenario had ended, the nursing students focused on identifying the main knowledge and skills provided by this simulation experience and aspects of the high-fidelity clinical simulation that could be useful for improving the care provided to patients with mental illnesses.

#### 3.2.1. Knowledge Acquired

When the students discussed what they had learned, they all agreed on two aspects: the management of these patients and relaxation and negotiation techniques.

- Management of mental health patients:

Experiencing a simulated situation with a mental health patient allowed the students to gain confidence in their interaction with these patients.

*Be calm with a severe patient. Know how to create a relationship of trust*.

(S8)

*I acquired more experience in these types of cases; how to talk to the patient and the presence of the nurses*.

(S21)

*Thanks to this scenario, we were able to discover the most effective way to deal with a mental health patient, and how to create a therapeutic relationship*.

(S26)

- Negotiation and relaxation techniques:

To successfully resolve the simulation scenario, the students had to delve into the knowledge and application of negotiation and relaxation techniques, which they valued positively.

*Knowing how to negotiate and knowing tools to manage emotions*.

(S4)

*Help the patient control his anger and impulses*.

(S13)

#### 3.2.2. How Does CS Help in the Care of Patients with Mental Disorders?

For students, the main potential benefit of high-fidelity clinical simulation in mental health is its ability to break down prejudices against these individuals; they also pointed out the confidence it provides them for their future clinical practice when facing scenarios that are very real life-like.

- Breaking down the stigma:

The social stigma of patients with mental disorders is one of the main barriers to their relationship with health professionals. Our students recognized that this simulation experience made them change their point of view about these individuals.

*In the beginning, I thought I would be more afraid, but in the end, you get to know them, and they would be unable to hurt a fly*.

(S19)

*Now I have good feelings, it feels as if I had dealt with them in the past*.

(S23)

*It normalizes mental health*.

(S27)

*It helps to end the stigma we find with mental health patients, as on many occasions, they see these patients as ‘impossible cases’*.

(S32)

- Provides confidence:

The students mentioned feeling more confident going into the simulated scenario when evaluating this experience as very useful for its application in clinical practice.

*As it was so real, the simulation helped us to prepare for situations that we will very likely find someday*.

(S22)

*It is very necessary knowledge for students, as it strengthens us and gives us the confidence needed for the development of emotional skills, which are fundamental in this field. In this scenario, we learn about what we do well, and what we could improve, and it is a very enriching experience*.

(S15)

## 4. Discussion

The need for the present study arose from the observation that during their education, a systematic tendency of nursing students was found to select clinical cases of patients with physical problems during the Practicum IV and V courses, associated with hospitalization, excluding the cases of patients with mental disorders. This led us to question if the opinions of the students could be marked by a certain social stigma toward these individuals or their lack of knowledge about their care. With this in mind, we designed a qualitative study to discover the attitudes, limitations, and conflicts of the students toward mental health patients and work on specific skills and competences through the use of clinical simulation sessions, which would allow them to improve their view of mental health by promoting changes in their attitudes and in the intention of working with mental health patients (treat, specialize, or work in the field) and by optimizing the attention and care given by nurses.

In general, the results obtained in the pre-CS interview confirm that many nursing students hold negative assumptions about mentally ill patients, with pre-conceived ideas, prejudices, and social stigmas [8]. In addition, these results also are coherent with other studies that have found nursing students can often lack confidence in communicating with these patients [9]. However, our participants changed and improved their perceptions toward mental health in the post-CS interview after the high-fidelity simulation training. This result is coherent with recent systematic reviews and meta-analyses which found that mental health-specific training may improve these perceptions [11], highlighting the usefulness of simulation training in mental health nursing education [12].

Specifically, below, we carry out a more detailed comparative analysis of the previous scientific evidence in this field. For this purpose, the discussion is structured according to the main categories that emerged from the participants’ discourses.

In the pre-CS interview, we observed that the nursing students’ opinions were marked by the social stigma of these individuals, qualifying the clinical cases with mental disorder patients as ‘complex, unstable, unpredictable, and little communicative’ and expressing training deficits in communication skills and a lack of clinical experience. Previous studies have also found attitudes of rejection and stigmatization among nursing professionals [6] and a fear of contact with the patient from students during their clinical practices [32], thus observing the need to promote inclusive education and to develop specific curricular interventions against the stigma [33]. In this sense, Carrara et al. [34] consider that anti-stigma interventions that involve social contact between primary health providers and patients with mental illnesses seem to be more efficient for reducing this stigma. However, our results and previous studies support education strategies without direct social contact, such as discussion groups or clinical simulation [35,36].

Moreover, our nursing students stated that they did not have sufficient knowledge to deal with the care of the patient with a mental illness. In this way, they created the dichotomy of mental versus physical health, since they understood that the physical is objectifiable and may be solved by knowing what to do, while for managing a mentally ill patient, some unspecified skills are needed, for which the biomedical model seems to be insufficient [37]. Even a strong academic curriculum is not enough to change stigmatized perceptions about mental illness, psychiatric care, and mental health nursing as a profession. In this sense, Martin et al. [7] consider that a general psychiatry course during nursing school is, by itself, unlikely to change biased views and should be enhanced by exposure to and interaction with people with lived experiences of mental illness.

After the high-fidelity simulation training, we found that the nursing students’ perceptions about mentally ill patients changed and improved in the post-CS interview. In this sense, previous studies have highlighted the usefulness of clinical simulation for the development of psychosocial evaluation skills, allowing the students to develop the necessary principles to offer safe and effective care to patients [35]. Specifically, the report after the simulation session is the cornerstone of the learning experience in a clinical simulation environment [27]. It allows both professors and students to re-examine the simulated case experience, share their mental model, and promote the rationale behind the clinical judgement [38].

In our study, once the simulation experience was completed, students focused on identifying the main knowledge skills provided by this simulation experience and aspects of the high-fidelity clinical simulation that could be useful for improving the care provided to patients with mental illnesses. In addition, our nursing students indicated that the simulation provided them with a realistic environment in which they were able to develop skills and manage clinical situations more independently, minimizing their feelings of insecurity and initial stigmatizing ideas, and to improve their learning deficiencies associated with communication skills. All of these findings confirm the results obtained in previous studies related to the use of clinical simulation methodology in mental health nursing education [8,9,12].

Finally, and from the point of view of educators, we believe that simulation-based education, when it is utilized in adequate conditions following the international best practices standards [26], correlates with significant effects on the knowledge, skills, and behaviors toward the patient with a mental illness [38]. In our case, the learning was based on the management of asevere mental health patient, using negotiation and relaxation techniques. In this sense, our nursing students highlighted the potential of high-fidelity simulation training in mental health education to break down their initial pre-conceived ideas (such as prejudices and social stigma) and provide them with confidence for facing their future clinical practice in real scenarios, aside from allowing them to delve into and acquire new knowledge [39].

### Limitations

Although the study reflects the perceptions of nursing students after staging a high-fidelity simulation case based on a mental health patient, it would be important to delve into these perceptions through a qualitative study at a greater scale, through the design of new simulated clinical cases with patients with other mental disorders, and through a larger sample of nursing students, as well as other studies with quantitative methodology to evaluate the effectiveness of this education intervention. Likewise, future studies based on the clinical simulation methodology should be conducted with nursing professionals. Thus, the clinical simulation teaching methodology should not only be expanded to education centers but also health centers, placing value on the acquisition of nursing competences needed for the adequate care of patients with mental disorders by nursing professionals and not only of students.

## 5. Conclusions

Considering the findings from our study, at first, the participants were insecure and reticent about working with mental health patients, classifying them as ‘complex and unpredictable patients’. However, after taking part in clinical simulation sessions, the students reported high levels of satisfaction with the simulation experience, stating that the simulation approach to these patients normalized mental health and helped them to be more prepared for clinical rotations.

In conclusion, experiences of active learning are necessary and efficient for the development of skills, provide participants with the confidence necessary for caring for patients with a mental disorder, and help break down the stigma of nursing professionals toward mental health patients.

## Figures and Tables

**Table 1:** Simulated scenario, appropriate NIC (Nursing Interventions Classification) interventions, and related nursing activities for its resolution.

Simulated Scenario	NIC Intervention	Nursing Activities
Juan. A 32-year-old man diagnosed with borderline personality disorder was admitted to the therapeutic community of mental health (a long-term hospital residency unit) 3 days ago. He does not want to get up and does not want to shower or eat because he does not want to be admitted. He feels sad, nervous, and angry because of the situation, and he is especially angry with his parents, whom he blames for the admittance.	(5270) Emotional support	Favor conversation over crying as a way to decrease emotional tension.
		Remain with the patient and provide safety during periods of higher anxiety.
		Comment on the emotional experience with the patient.
		Provide help in the making of decisions.
		Encourage the patient to express his feelings of anxiety, anger, or sadness.
	(4640) Anger control assistance	Support the patient in the practice of anger control strategies and their adequate manifestation.
		Instruct the patient about measures that provide calmness (resting and deep breathing).
		Identify the consequences of the inadequate expression of anger.
		Use an approach that is serene and safe.
		Comment on the changes in lifestyle that could be necessary to avoid future complications and/or control the disease process.

**Table 2:** Interview guide.

Pre-clinical simulation phase	Explain what difficulties you expect to find when you address the case.
	According to you, what features or characteristics define patients with mental disorders?
	What perceptions and feelings or emotions do you feel when you have to care for a patient with a mental disorder?
Post-clinical simulation	What knowledge and/or skills do you think you have acquired after this experience?
	What usefulness do you see in the use of clinical simulation in mental health nursing?

**Table 3:** List of categories and subcategories identified.

Interview	Category	Sub-Category
Pre-clinical simulation	What difficulties I will find.	Complex case Not knowing
	Perceptions of patients with a mental disorder.	The prejudices; the stigma Like another patient
	Emotions felt with regard to a patient with a mental disorder.	Fear Insecurity
	What is lacking in my training.	Communication skills Clinical experience Unspecified training
Post-clinical simulation	Knowledge acquired.	Negotiation and relaxation techniques Management of mental health patient
	How does CS help in the care of patients with mental disorders?	Breaking the stigma Provides confidence

## Data Availability

The data presented in this study are available on request from the corresponding author.

## Public Involvement Statement

No public involvement in any aspect of this research.

## Guidelines and Standards Statement

QOREQ: (Consolidated criteria for Reporting Qualitative research) checklist. The use of high-fidelity simulation lets nursing students become closer to mental health patients, overcoming their fears, and normalizing mental disorders. This training gave students the necessary confidence when caring for individuals with mental disorders, breaking away from the stigma toward these patients. Therefore, it is considered that the realization of clinical simulation sessions in mental health nursing education has a positive effect on their future healthcare practice, since it allows students to share their mental model, analyze the reasoning behind clinical judgment, and detect the possible influence of previous prejudices about mental illness in their clinical decision.

## References

[1] Motrico E., Salinas-Perez J.A., Rodero-Cosano M.L., Conejo-Cerón S. (2021). Editors’ comments on the special issue “social determinants of mental health”. Int. J. Environ. Res. Public Health.

[2] World Health Organization (2022). World Mental Health Report: Transforming Mental Health for All. https://www.who.int/publications/i/item/9789240049338.

[3] Goffman E. (1968). Stigma: Notes on the Management of Spoiled Identity.

[4] Angermeyer M.C., Dietrich S. (2006). Public beliefs about and attitudes towards people with mental illness: A review of population studies. Acta Psychiatr. Scand..

[5] Eksteen H.C., Becker P.J., Lippi G. (2017). Stigmatization towards the mentally ill: Perceptions of psychiatrists, pre-clinical and post-clinical rotation medical students. Int. J. Soc. Psychiatry.

[6] Valverde-Bolivar E., García-Arenas J.J., López-Pelegrin I., Pérez-Gómez L., Muñoz-López M., Simonelli-Muñoz A.J. (2022). The stigma of mental health professionals towards users with a mental disorder. Actas Esp. Psiquiatr..

[7] Martin A., Krause R., Chilton J., Jacobs A., Amsalem D. (2020). Attitudes to psychiatry and to mental illness among nursing students: Adaptation and use of two validated instruments in preclinical education. J. Psychiatr. Ment. Health Nurs..

[8] Ogard-Repal A., De Presno A.K., Fossum M. (2018). Simulation with standardized patients to prepare undergraduate nursing students for mental health clinical practice: An integrative literature review. Nurse Educ. Today.

[9] Vandyk A.D., Lalonde M., Merali S., Wright E., Bajnok I., Davies B. (2018). The use of psychiatry-focused simulation in undergraduate nursing education: A systematic search and review. Int. J. Ment. Health Nurs..

[10] Heim E., Henderson C., Kohrt B.A., Koschorke M., Milenova M., Thornicroft G. (2019). Reducing mental health-related stigma among medical and nursing students in low- and middle-income countries: A systematic review. Epidemiol. Psychiatr. Sci..

[11] Giralt Palou R., Prat Vigué G., Tort-Nasarre G. (2020). Attitudes and stigma toward mental health in nursing students: A systematic review. Perspect. Psychiatr. Care.

[12] Piot M.A., Dechartres A., Attoe C., Romeo M., Jollant F., Billon G., Cross S., Lemogne C., Layat Burn C., Michelet D. (2022). Effectiveness of simulation in psychiatry for nursing students, nurses and nurse practitioners: A systematic review and meta-analysis. J. Adv. Nurs..

[13] Cremonini V., Pagnucci N., Giacometti F., Rubbi I. (2018). Health care professionals attitudes towards mental illness: Observational study performed at a public health facility in northern Italy. Arch. Psychiatr. Nurs..

[14] Siqueira S.R., Abelha L., Lovisi G.M., Sarução K.R., Yang L. (2017). Attitudes towards the mentally ill: A study with health workers at a university hospital in Rio de Janeiro. Psychiatr. Q..

[15] Williams A.A. (2020). The next step in integrated care: Universal primary mental health providers. J. Clin. Psychol. Med. Settings.

[16] Ortiz-Sandoval I., Martínez-Quiles M.D., López-Pérez J., Simonelli-Muñoz A.J. (2022). Triggers of agitation in psychiatric hospitalization ward according to professional experience questionnaire. Int. J. Environ. Res. Public Health.

[17] Patterson C., Moxham L., Brighton R., Taylor E., Sumskis S., Perlman D., Heffernan T., Hadfield L. (2016). Nursing students’ reflections on the learning experience of a unique mental health clinical placement. Nurse Educ. Today.

[18] Wedgeworth M.L., Ford C.D., Tice J.R. (2020). “I’m scared”: Journaling uncovers student perceptions prior to a psychiatric clinical rotation. J. Am. Psychiatr. Nurses Assoc..

[19] García-Mayor S., Quemada-González C., León-Campos Á., Kaknani-Uttumchandani S., Gutiérrez-Rodríguez L., Del Mar Carmona-Segovia A., Martí-García C. (2021). Nursing students’ perceptions on the use of clinical simulation in psychiatric and mental health nursing by means of objective structured clinical examination (OSCE). Nurse Educ. Today.

[20] Jiménez-Rodríguez D., Belmonte García M.T., Santillán García A., Plaza del Pino F.J., Ponce-Valencia A., Arrogante O. (2020). Nurse training in gender-based violence using simulated nursing video consultations during the COVID-19 pandemic: A qualitative study. Int. J. Environ. Res. Public Health.

[21] Sandelowski M. (2000). Whatever happened to qualitative description?. Res. Nurs. Health.

[22] De la Cuesta C. (2006). Naturaleza de la investigacióncualitativa y sucontribución a la práctica de enfermería. Metas Enfermería.

[23] Tong A., Sainsbury P., Craig J. (2007). Consolidated Criteria for Reporting Qualitative Research (COREQ): A 32-Itemchecklist for Interviews and Focus Groups. Int. J. Qual. Health Care.

[24] American Psychiatric Association (2022). Diagnostic and Statistical Manual of Mental Disorders: DSM-5-TR.

[25] Butcher H.K., Bulechek G.M., Dochterman J.M., Wagner C.M. (2018). Nursing Interventions Classification (NIC).

[26] INACSL Standards Committee (2016). INACSL Standards of Best Practice: Simulation^SM^ Simulation design. Clin. Simul. Nurs..

[27] Rudolph J.W., Raemer D.B., Simon R. (2014). Establishing a safe container for learning in simulation the role of the presimulation briefing. Simul. Healthc..

[28] INACSL Standards Committee (2016). INACSL Standards of Best Practice: Simulation^SM^ Debriefing. Clin. Simul. Nurs..

[29] Phrampus P.E., O′Donnell J.M., Levine A.I., De Maria S., Schwartz A.D., Sim A.J. (2013). Debriefing using a structured and supported approach. The Comprehensive Textbook of Healthcare Simulation.

[30] Doyle L., McCabe C., Keogh B., Brady A., McCann M. (2020). An overview of the qualitative descriptive design within nursing research. J. Res. Nurs..

[31] Lincoln K., Guba E. (1986). But is it rigorous? Trustworthiness and authenticity in naturalistic evaluation. New Dir. Program Eval..

[32] Itzhaki M., Meridan O., Sagiv-Schifter T., Barnoy S. (2017). Nursing students’ attitudes and intention to work with mentally ill patients before and after a planned intervention. Acad. Psychiatry.

[33] Zhang Z., Sun K., Jatchavala C., Koh J., Chia Y., Bose J., Li Z., Tan W., Wang S., Chu W. (2019). Overview of stigma against psychiatric illnesses and advancements of anti-stigma activities in six asian societies. Int. J. Environ. Res. Public Health.

[34] Carrara B.S., Fernandes R., Bobbili S.J., Ventura C. (2021). Health care providers and people with mental illness: An integrative review on anti-stigma interventions. Int. J. Soc. Psychiatry.

[35] Calohan J., Pauli E., Combs T., Creel A., Convoy S., Owen R. (2016). Using simulation in a psychiatric mental health nurse practitioner doctoral program. J. Prof. Nurs. Off. J. Am. Assoc. Coll. Nurs..

[36] Hutson E., Zeno R. (2021). Clinical competence for youth suicide: Use of simulation in pediatric and psychiatric-mental health nurse practitioner programs. J. Psychosoc. Nurs. Ment. Health Serv..

[37] Rodríguez-Almagro J., Hernández-Martínez A., Rodríguez-Almagro D., Quiros-García J.M., Solano-Ruiz M., Gómez-Salgado J. (2019). Level of stigma among spanish nursing students toward mental illness and associated factors: A mixed-methods study. Int. J. Environ. Res. Public Health.

[38] Abulebda K., Auerbach M., Limaiem F. (2021). Debriefing Techniques Utilized in Medical Simulation. StatPearls.

[39] Felton A., Wright N. (2017). Simulation in mental health nurse education: The development, implementation and evaluation of an educational innovation. Nurse Educ. Pract..

